# Perceived Service Quality in HRI: Applying the SERVBOT Framework

**DOI:** 10.3389/frobt.2021.746674

**Published:** 2021-12-13

**Authors:** Isha Kharub, Michael Lwin, Aila Khan, Omar Mubin

**Affiliations:** ^1^ School of Business, Western Sydney University, Sydney, NSW, Australia; ^2^ School of Computer, Data and Mathematical Sciences, Western Sydney University, Sydney, NSW, Australia

**Keywords:** human-robot interaction, service quality, SERVQUAL, concierge, retail

## Abstract

Services are intangible in nature and as a result, it is often difficult to measure the quality of the service. In the service literature, the service is usually delivered by a human to a human customer and the quality of the service is often evaluated using the SERVQUAL dimensions. An extensive review of the literature shows there is a lack of an empirical model to assess the perceived service quality provided by a social robot. Furthermore, the social robot literature highlights key differences between human service and social robots. For example, scholars have highlighted the importance of entertainment value and engagement in the adoption of social robots in the service industry. However, it is unclear whether the SERVQUAL dimensions are appropriate to measure social robot’s service quality. The paper proposes the SERVBOT model to assess a social robot’s service quality. It identifies, reliability, responsiveness, assurance, empathy, and entertainment as the five dimensions of SERVBOT. Further, the research will investigate how these five factors influence emotional engagement and future intentions to use the social robot in a concierge service setting. The model was tested using student sampling, and a total of 94 responses were collected for the study. The findings indicate empathy and entertainment value as key predictors of emotional engagement. Further, emotional engagement is a strong predictor of future intention to use a social robot in a service setting. This study is the first to propose the SERVBOT model to measure social robot’s service quality. The model provides a theoretical underpinning on the key service quality dimensions of a social robot and gives scholars and managers a method to track the service quality of a social robot. The study also extends on the literature by exploring the key factors that influence the use of social robots (i.e., emotional engagement).

## Introduction

Traditionally, services were solely provided by humans to other humans. However, with the advancement in technology, social robots are increasingly being used in the service sector to fulfil a service (Wirtz et al., 2018; Chiang and Trimi, 2020). Rapid development in the field of digital technologies such as artificial intelligence, the Internet of Things (iot), mobile and cloud technology, and social robotics are transforming the service sector and changing customer service expectations and experiences (Huang and Rust, 2018; Wirtz et al., 2018; Pavon et al., 2020). Additionally, the COVID-19 pandemic proved to be a catalyst in advancing the robotics ecosystem and driving robotic adoption (Tung, 2020; Yang et al., 2020; Zeng et al., 2020). During the COVID-19 pandemic, social robots were successfully deployed in hotels, retail stores, hospitals, airports, and public spaces, proving the importance and usefulness of deploying robots in a wide range of services and industries. Social robots proved to be useful for preventing cross infections through contactless services (Pani et al., 2020). They also provided therapeutic and entertainment for quarantined patients and the vulnerable (Aymerich-Franch and Ferrer, 2020).

The term robot was first coined by Karel Capek in 1920 and was later used in short book written by Isaac Asimov in the 1930s (Hegel et al., 2008; Hegel et al., 2009). The word “robot” originated from the word “robota” which means forced labour in Czech (Jordan, 2019). However, robots have evolved from being just dumb machines who perform repetitive tasks to being highly intelligent robots that look and act like humans. (Lanfranco et al., 2004). Service robots are “system-based autonomous and adaptable interfaces that interact, communicate, and deliver services to an organisations customers” (Wirtz et al., 2018). As per Engelhart, service robots are systems that can function as smart, programmable tools, that can sense, think, and act to benefit or enable humans or extend/enhance human productivity (Engelhardt et al., 1992). Service robots can be 1) virtual or have a physical presentation 2) humanoid or non-humanoid 3) and can perform both cognitive-analytical and emotional-social tasks (Wirtz et al., 2018). When service robots are used in a frontline service setting, they can be called social robots as they interact and co-create value with their customers during the interaction (Wirtz et al., 2018; Čaić et al., 2019). Social robots were specifically designed for the interaction between robots and humans and support human-like interactions (Hegel et al., 2008). It is important to note that during the service encounter, service robots can create a degree of Automated Social Presence (ASP), making the customer feel like they are in the presence of another social entity (Van Doorn et al., 2017).

Social robots are increasingly being used in the structured and repetitive environment, services sector (receptionists in hotels; museum tour guides; teaching assistants in education) and for personal use (companions in aged care; zoomorphic robots) (Hegel et al., 2008; Louie et al., 2014; Mejia and Kajikawa, 2017; Tussyadiah and Park, 2018; Wirtz and Zeithaml, 2018). Due to the advancement in artificial intelligence (AI), robots have been equipped with “social intelligence”. This gives robots to be socially aware and equip them with the ability to decipher emotional signals and react in a human-like manner (Breazeal, 2003; Lazzeri et al., 2013). Humanoid robots are a form of social robots that can exhibit social behaviour and create human-like interactions. They make decisions autonomously based on the data they receive from sensors and other sources and adapt to different situations accordingly (Wirtz et al., 2018).

However, due to technological limitations it’s difficult for robots to work independently, especially in a situation that requires intuition, judgment, and empathy (Huang and Rust, 2018). The gap between the service provided by humans and by robots is still large, sometimes large enough to render them useless (Chiang and Trimi, 2020). For example, a well-known hotel chain “Henn-na hotel” initially deployed robot staff to replace human staff. However, due to the robot’s poor service quality, humans had to be recalled to replace the robot staff (Ryall, 2019). As per Computers Are Social Actions (CASA) paradigm, humans treat computers as social entities and consequently, the social robots will need to be equipped with the same requirements as a human service agent (Nass et al., 1994; Niculescu et al., 2013). Amelia et al. (2021) found that participants interacted and engaged with the social robot in the same way as they would with their partners in a human-human interactions. Additionally, the participants gave social cues to the social robot such as “Thank You” or “Goodbye” (Amelia et al., 2021). More importantly, the participant’s interaction with the social robot influenced their perception of the company (Amelia et al., 2021). Therefore, a social robot’s performance will impact the user’s perception of the service quality and subsequently user’s behavioural intentions (Bartneck et al., 2009).

The SERVQUAL model has been widely used to measure service quality in a number of contexts and cultural settings including, tourism (e.g., Shafiq et al., 2019), healthcare (e.g Pekkaya et al., 2017), banking (e.g., Raza et al., 2020), education (e.g., Banahene et al., 2017), and government (e.g., Ocampo et al., 2019). The five dimensions of SERVQUAL (reliability, responsiveness, assurance, empathy and tangibles) have been shown to reliably predict service quality of human frontline service employees (e.g., Parasuraman et al., 1988). However, social robots are very different from humans in service delivery and entertainment value is integral in HRI (Morita et al., 2020). Due to the nature of social robots, the original SERVQUAL model is inadequate for measuring service quality (Morita et al., 2020). For example, scholars in social robotics have highlighted that engagement and entertainment are key to the adoption of the technology (e.g., Coulter et al., 2012; Schodde et al., 2017; Liu et al., 2019). A lack of empirical data (Čaić et al., 2019; Chiang and Trimi, 2020; Lu et al., 2020) and a well-defined framework in this area means it is very difficult to identify the variables that are critical to measuring the social robot’s service quality (Chiang and Trimi, 2020). To date, only one study has attempted to examine this phenomenon using the SERVQUAL framework (Morita et al., 2020). However, the study failed to adapt the critical factors that are important in the evaluation of the service quality dimensions (e.g., entertainment value and emotional engagement). As mentioned above, social robots are very different from humans in service delivery. As such it is unclear whether the SERVQUAL five dimensions are relevant or whether other dimensions should be added to measure the social robot’s service quality.

An extensive review of the literature shows a lack of quantitative analysis that examines social robot’s service quality in human-robot interaction or the business literature. The paper attempts to fulfil these research gaps and provide a framework to measure social robot’s service quality. This exploratory research will empirically examine the effects of a social robot’s service quality on user engagement and behavioural intentions. Due to limitations in technology and use of service robots being a new phenomenon, this empirical research will attempt to identify the potential antecedents of emotional engagement (Tuomi et al., 2021). Moreover, the study attempts to understand the importance of the service quality dimensions in robot-induced service delivery. The most relevant studies in the area focus on chatbots, and these studies suggests SERVQUAL can accurately measure social robot’s service quality (Pavon et al., 2020). Thus, the study will provide key insights into the usage of social robots in a service setting by using a well-established theory.

## Service Quality

Service quality is frequently studied in service marketing literature and many researchers have tried to understand and identify service quality in the last 4 decades. To compete successfully in future and to gain a competitive advantage, businesses will have to develop the quality of their service (Gronroos, 1984; Parasuraman et al., 1988). The quality of products and services is seen as a strategic variable to achieve efficiency and effective in business operations (Babakus and Boller, 1992). However, different researchers have defined service quality differently. For example, according to Lehtinen and Lehtinen (1991), service quality is produced during a interaction between the customer and the elements of service organisation such as contact person/s. Whereas as Parasuraman et al. (1998), defined service quality as the difference between customer’s expectation of a service and perceptions of the service quality. As per Carman (1990), the reason behind different definitions is because the conceptualisation and measurement of service quality is an elusive concept due to the intangibility, simultaneous production and consumption of a service, and the difference between mechanistic and humanistic quality (Carman, 1990:33).

Perceived service quality is an overall judgment of a service that contributes to a range of positive outcomes for a firm (Cronin and Taylor, 1992). Scholars have suggested that service quality stems from a comparison of what customers feel a company should offer (expectations) with the company’s actual performance (Parasuraman et al., 1988) Traditionally, service quality has been conceptualised for people-delivered services.

### SERVQUAL Modifications

However, as technological innovations continue to grow, a critical component of customer-firm interactions is driven by the rise of self-service and humanoid technologies (Meuter et al., 2000). With increasing proliferation of e-commerce and declining face to face interactions, the SERVQUAL model was modified. For example, the traditional five SERVQUAL dimensions did not adequately measure customers interaction with a website (Ladhari, 2009). Consequently, E-S-QUAL was developed to measure e-SQ and it was shown to be a highly applicable for the online service environment.

However, SERVQUAL has been subjected to a number of theoretical and operational criticisms (Buttle, 1996). Some of these criticisms revolve around inapplicability of the SERVQUAL dimensions across different industries and some criticise the efficacy of SERVQUAL model itself.

There is a consensus in service marketing literature that service quality is a multi-dimensional or multi-attribute construct (Kang and James, 2004). According to Parasuraman et al. (1998), service quality can be evaluated based on functional quality characterised by Reliability, Responsiveness, Empathy, Tangibles, and Assurance (Parasuraman et al., 1988). These dimensions are a part of scale called SERVQUAL. SERVQUAL was conceptualised to measure service quality and has proved to be a reliable, widely applicable, and concise instrument to measure service quality. Managers can evaluate a firm’s perceived service quality using a multi-item scale with above mentioned five dimensions (Parasuraman et al., 1988).

However, Babakus and Boller (1992) explained that service quality can be factorially complex in certain industries, and very simple and unidimensional in others. Thus, the dimensions are dependent on the services being offered. There is no real consensus on which dimensions are relevant for the service quality (Philip and Hazlett, 1997). For example, the hospitality industry research employed 40 items (Carman 1990), while the car service studies employed 48 items (Bouman and Van der Wiele 1992). Therefore, it has been suggested in the literature that context specific modifications must be made to increase the relevancy of SERVQUAL scale or measures should be designed for specific service industries (Babakus and Boller, 1992). Cronin and Taylor (1992) criticised the SERVQUAL framework for using expectations as current performance, not expectations, best reflects a customer’s perception of service quality (Cronin and Taylor, 1992). This was also confirmed by Quester et al. (2015) who found SERVPERF, performance only measure, to be better than SERVQUAL (disconfirmation measure) (Quester et al., 2015). However, Parasuraman et al., 1994) and Bolton and Drew (1991) found that the disconfirmation model had greater diagnostic ability and predictive power (Bolton and Drew, 1991; Parasuraman et al., 1994).

To evaluate the service quality of social robots, SERVQUAL needs to be modified as it is inadequate for measuring service quality of social robots (Morita et al., 2020). The service quality provided by social robots is impacted not just by their technical capabilities but also customer’s expectations (Chiang and Trimi, 2020). It is critical that human interaction, perceptions, motivations and emotional reactions are understood and evaluated (Piçarra and Giger, 2018). Further, these perceptions are direct predictors of service quality and engagement with users (Diaz et al., 2011; Anzalone et al., 2015).

Further, a category of user experience is described as engagement and this has shown to have a direct impact on user’s behavioral intention (Anzalone et al., 2015). For successful use of social robots, customer inputs and cocreation are necessary to ensure robots are fulfilling customer wants and expectations of service quality (Baisch et al., 2017; Čaić et al., 2018). More significantly, acceptability is increased when the robot is entertaining (Whelan et al., 2018). Thus, the study proposes entertainment as an additional dimension to the SERVQUAL framework. This was further supported by other studies that showed “entertaining robots” had a positive influence on the customers’ behaviour (Morita et al., 2020). This. this study will explore the influence of these six dimensions on emotional engagement.

### Types of Service Quality

Service is assessed on two main quality dimensions, technical and functional quality (Lehtinen and Lehtinen, 1991). Technical quality is referred to as what the customer receives as an *outcome* of the service process which is sometimes a tangible output such as a meal (Lehtinen and Lehtinen, 1991). It may also refer to an intangible output such as information received from the concierge.

On the other hand, customers are also likely to evaluate the service based on its functional quality (Gronroos, 1984), also referred to as the interactive quality (Lehtinen and Lehtinen, 1991). This type of quality is derived from the interaction between the service provider and customers. Interactive quality refers to the process in which the technical component of the service is transferred to the customer. This may also involve customers’ participation in the service delivery process (Lehtinen and Lehtinen, 1991).

### Social Robots and Service Quality (SQ)

Increasingly, robots are being employed to carry out frontline tasks, such as guiding shoppers through stores (Rafaeli et al., 2017), assisting clients in opening bank accounts (Byford, 2015), and serving customers in restaurants (Nguyen, 2016). This growing use of technology by a range of service providers has sparked academic interest across many disciplines (e.g., (Mubin et al., 2016). However, there is limited empirical research to evaluate customers’ perceptions of a robot-delivered service quality (Choi et al., 2020; Shin and Jeong, 2020; Zhong et al., 2020).

#### Types of Social Robots: Based on Appearance

It is important to note that all robots are not social robots and not all social robots are humanoid robots (Zhao, 2006). The appearance of social robots is integral when assessing its performance and appropriateness in a particular context (Lohse et al., 2007). Fong et al. (2003) proposed four types of robot based on robot morphology: zoomorphic robots, functional robots, caricature robots and anthropomorphic robots (Fong et al., 2003).

Zoomorphic robots are social robots that resemble animals such as dogs, cats or seals (Klamer and Allouch, 2010; Takayanagi et al., 2014). Zoomorphic robots such as Paro (image a), a baby harp seal, is used to stimulate users and connect with their prior experience by evoking happiness and caring emotions that are generated while interacting with pets. It is specially designed for therapeutic purposes in older adults, paediatric and autistic patients (Lane et al., 2016).

Functional robots are designed with the purpose of fulfilling operational objectives (Fong et al., 2003). They are designed to fulfil a given tasks or function such as Roomba or PackBot (Veloso et al., 2015). Their appearance leans towards mechanical aspects, purely directed by operational objectives fulfilment (Fong et al., 2003).

Caricature robots are designed to look like cartoons. They do not need to be realistic in order to appear believable. In fact, they are designed to show humanoid motions in exaggerated ways (Sebastian et al., 2015).

Anthropomorphic robots are structurally and functionally similar to human beings. They are robots with human-like appearance and behave in a human-like manner (Phillips et al., 2018). Anthropomorphism can defined as “the tendency to imbue the real or imagined behaviour of non-human agents with humanlike characteristics, motivations, intentions or emotions’ (Epley et al., 2007) In Human-Robot Interaction (HRI), it has been found that anthropomorphism is a strong determinant of user preference and perceived trust (van Pinxteren et al., 2019). Anthropomorphic robots are humanoid or human-shaped robots. they can be defined as “human-made entities (robotic), that interact with humans (social) in a human-like way (humanoid)” (Zhao, 2006). In short, humanoid robots are anthropomorphized. Anthropomorphism has great impacts on technology adoption rate, service quality and service experience (Yoganathan et al., 2021). Studies have shown that guests have higher social expectations of anthropomorphic robots than zoomorphic, caricature and functional robots (Choi et al., 2020). This is because humanoid robots offer more meaningful interaction in HRI (Ziemke and Thill, 2014). This is corroborated with Tussyadiah and Park (2018) study that found anthropomorphism to be the key in influencing user adoption.

#### Challenges in Measuring Service Quality of Social Robots

The investigation of service quality, in the context of social robots, is important from two key perspectives. First, in a robot-human interaction it is similar to other forms of technology, humanoid agents such as robots trigger both positive and negative feelings in users (Englis, 1990; Wiese et al., 2017). Users may simultaneously present views (i.e., perceptions, beliefs, feelings, motivations) that are both favourable and unfavourable. Researchers agree that the co-existence and balance between these forces of attraction and repulsion determine the individual’s likelihood to adopt–and consequently–evaluate–service delivery by robots (Bishop et al., 2019). Consumers with highly positive views of technology are likely to be receptive to robot-based services. On the other hand, users with a highly negative view of technology (e.g., individuals who feel discomfort or insecurity) might be resistant towards such services (Ferreira et al., 2014; Wiese et al., 2017). It is well-accepted that not all users are equally ready to embrace technology-assisted services (Yen, 2005). Therefore, in line with Parasuraman et al. (1998) and Colby and Parasuraman (2001) findings, it is expected that different users will evaluate technology-based services in different ways.

The second challenge in a robot-delivered service is the knowledge that competitors may easily mimic the technical quality of service provision, particularly as some of the social robots used in service settings are acquired off the shelf and they operate using open source software (Gronroos, 1988; Bartneck et al., 2020). This means that it would be simple for competing retail outlets or restaurants to provide the features enabling particular services through the use of such robots. However, it is far more difficult for competitors to replicate interactive service quality. The interactive quality dimension refers to the actual interaction which takes place between the customers and the frontline staff members (Lehtinen and Lehtinen, 1991). In a study by Nakanishi et al. (2020), it was found that heart-warming interactions can enhance customer’s overall satisfaction with the hotel services (Nakanishi et al., 2020). This was determined by using qualitative and preference based questionnaire data. These heart-warming interactions are behaviours and attitudes that can create feelings of interpersonal warmth through a smile, a greeting or eye contact (Nakanishi et al., 2020). This is a constant challenge for robot designers and operators to ensure that the development of an embodied agent is not just limited to attractive physical characteristics. In fact, any agent involved in service delivery must exhibit naturalistic behaviour and appropriate emotional engagement which is highly valued by the customer (Woods, 2006; Cavallo et al., 2018). There is a need for continued research to understand public perceptions about evolving impacts of social robots in society (de Kervenoael et al., 2020).

Additionally, there is no scale available to evaluate the service quality of social robots. Although scales like Goodspeed questionnaire exist, they are primarily used by creators and developers in their development journey (Bartneck et al., 2009). Even though theoretical frameworks have been used in multiple fields, the frameworks that are hospitality-specific are still lacking (Pan et al., 2015; Ivanov et al., 2017). There is a call for more theoretical and methodological framework to understand HRI better, particularly to enhance user experiences (Bartneck et al., 2009; Tonkin et al., 2018; Ivanov et al., 2019). Most of the current work (e.g., Kamei et al., 2011; Niemelä et al., 2017; Niemelä et al., 2019; Nakanishi et al., 2020; Amelia et al., 2021) around robots in retail focuses on considerably light (or non-empirical) modes of evaluation (such as self-made questionnaires, interviews and acceptance surveys), with the focus on exploratory and technology based interventions.

Service quality, a fundamental concept of customer’s service experience construct, is considered to be a useful tool to measure and examine various aspects of Human-Robot Interaction (Choi et al., 2020). However, to the best of authors knowledge, no attempt has been conducted to apply the modified SERVQUAL framework to understand the impact of service quality of social robots on user’s engagement and intention to use. Industry practitioners and academics have called for more research on how the social robots influence customers perception of overall service quality (Choi et al., 2020; Lu et al., 2020). SERVQUAL in its original form is inadequate for measuring service quality of social robots (Morita et al., 2020). This is because service quality of a robot is very different than that of humans and entertainment value is highly regarded in HRI (Morita et al., 2020). Mick and Fournier (1998) found that technology can induce positive and negative feelings simultaneously, and therefore SERVQUAL scale needs to be modified to understand which service dimensions or robot’s attributes induce what feelings. Even though a study by Choi et al. (2020) was conducted to examine how hotel guests perceive the quality of service provided by hotel staff and service robots, the study is limited as they used *images* of hypothetical encounters between the robot and the staff instead of a real time robot-human interaction. Further, it has been acknowledged in the literature that service quality should be measured after customers have interacted with the services (Morita et al., 2020). Therefore, the study will examine the service quality perceptions after the participants have interacted with the social robot staff in real time. This will reflect the actual guest experience of interacting with social robots.

Chiang and Trimi (2020) explored the service quality provided by robots using the SERVQUAL framework after the guests experienced the service. However, their study did not use a social robot and researchers have acknowledged that users have higher expectations of anthropomorphic robots or humanoid robots (Ziemke and Thill, 2014; Choi et al., 2020). Anthropomorphism significantly influences customers adoption intention and customers have higher social expectations from them (Ziemke and Thill, 2014; Tussyadiah and Park, 2018). Social robots have anthropomorphic characteristics which helps elicit joy and sympathy (Hegel et al., 2008). Secondly, this was not a comparative study where the service quality of service robots was compared with that of a human. Therefore, it failed to provide a comparison and failed to indicate how the robot compares to the human service quality. Morita et al. (2020) used humanoid robots in a multi-robot café to evaluate the service quality. Their questionnaire items were based on SERVQUAL and include entertainment. However, they evaluated the service quality and customer satisfaction, not emotional engagement. Emotional engagement is critical for the adoption of the technology and their research failed to address how this variable influences the social robot’s service quality.

It is important to understand customer’s experience and views about their interaction with social robots in frontline service settings (Amelia et al., 2021). The ultimate success of social robots in service settings depends on the engagement and satisfaction of customers (Bartneck et al., 2009).

## SERVBOT Framework

This research extends the original SERVQUAL framework (Parasuraman et al., 1988) to a service scenario with a social robot. The SERVQUAL framework is recognised as a rigorous model and has been applied across a number of service industries to measure service quality from the customers’ perspective (Brown et al., 1993). This study uses the original dimensions from the SERVQUAL model: reliability, responsiveness, assurance, and empathy. As highlighted earlier, entertainment is a critical driver in the adoption of social robots (Schodde et al., 2017). Thus, the “entertainment” dimension has been added to the SERVBOT model. Further, the “tangibles” dimension in SERVQUAL is defined as “physical facilities, equipment and communication material”. The research uses Pepper the robot voice command to communicate to the customer. As such the tangible dimension is not appropriate for the study and it is removed from the analysis. All SERVBOT dimensions are discussed below:

### Reliability

Reliability is the ability (of the social robot) to perform the promised service dependably and accurately’ (modified from Parasuraman et al., 1988). According to research, reliability is important for favourable evaluations in information systems. For a chatbot, it was found that reliability was the strongest determinant of perceived usefulness (Meyer-Waarden et al., 2020). In terms of human-robot interaction, reliability is whether service robot reliably performed the committed services (Chiang and Trimi, 2020). According to research, reliability is important for favourable evaluations in information systems. In a situation where a robot is used to provide users with information, this dimension refers to the reliability of information being provided (Xifei and Jin, 2015) and performing the promised service accurately (Parasuraman et al., 1988). In the robot café study, customers evaluated the reliability aspect highly and this increasing their willingness to engage with the robot more in a service setting (Morita et al., 2020). Therefore, it is predicted that reliability will have a positive impact on emotional engagement. See H1a below for the hypothesis.

### Responsiveness

Responsiveness is the willingness to help users, provide prompt service and timely responses. While in a usual service firm it may refer to businesses’ quick response to phone or email queries (Yang and Fang, 2004), in a robot-concierge situation, it may look at how promptly is the robot able to handle customer enquiries. As responsiveness increases, perceived service quality increases (Asubonteng et al., 1996). A social robot should not just be reactive but also proactive by not just responding to external events but also voluntarily providing information when necessary (Salichs et al., 2006). Limited responsiveness and contingency can decrease the users trust and feelings of closeness (Fox and Gambino, 2021). It is the responsiveness of social robots’ and their immediacy of actions towards specific tasks that affect how, where and when visitors would interact (or not) with them. A technology that does not respond to visitors cannot survive in today’s hypercompetitive marketplace (de Kervenoael et al., 2020). A responsive robot will be seen as more competent, sociable and attractive (Birnbaum et al., 2016). Thus, it is predicted that higher level of responsiveness will result in higher levels of emotional engagement (See H1b below).

### Assurance

Assurance is the knowledge and courtesy of the service provider (e.g., the robot) and its ability to convey trust and confidence. It leads to long term relationships and loyalty (de Kervenoael et al., 2020). In hospitality and tourism, service providers are expected to be specialists in the type of service they provide and to adapt to any new changes involving robots supporting humans (de Kervenoael et al., 2020). For social robots, it is the ability of the service robot to perform task with expertise, politeness, and trust (Chiang and Trimi, 2020). It refers to the robots’ ability to create feelings of trust and confidence among customers (Ivkov et al., 2020). Assurance in hospitality and tourism is about maintaining and enhancing the quality of service provided by robots to customers. In some cultural contexts, this has been identified as the most important dimension of service quality (Raajpoot, 2004). In a robot-interaction scenario, assurance could refer to making users confident of their physical safety. Similar to an online context, it refers to assuring customers of security or confidentiality during communication. Therefore, it is predicted that higher level of assurance will result in higher levels of emotional engagement (See H1c below).

### Empathy

Out of all the service quality dimensions, empathy has been studied the most in human-robot interaction literature. Empathy is recognised as a basic human trait (e.g., Klotz, 2018) but is also considered essential in a “socio-emotional” machine (Weber, 2005:209) for its acceptance by users. It is the driver of trust, loyalty, and long-term relationships (de Kervenoael et al., 2020). In HRI, empathy is providing care and personal attention to customers during the service (Chiang and Trimi, 2020).

Within service management, empathy is understood to be a fundamental skill required for successful interactions between social robots and users, for example, a good receptionist should not just be able to communicate effectively but also show empathy and provide help (Niculescu et al., 2013). It is a driver of trust, loyalty and long–term relationships because empathy requires all the parties involved must understand various positions, stands, requirements and needs to priortise tasks and actions from the customer’s perspectives. (Parasuraman et al., 1988). It leads to the creation and the development of social relationships by increasing fondness, similarity and affiliation. Therefore, social robots should have similar characteristics (Niculescu et al., 2013). Empathetic capabilities are important for long term HRI (Leite et al., 2012). This is corroborated by the Social Cognition Perspective which highlights the importance of Perceived Warmth in human-robot interactions (Čaić et al., 2019). Furthermore, this is line with Computers Are Social Actors paradigm which suggests that users expect the same social rules of human-human interaction to human-robot interaction (Nass et al., 1994).

Brave et al. (2005), found that modeling empathetic emotions in agents increased their positive ratings for likeability and trustworthiness (Brave et al., 2005). Empathetic agents were also perceived to be as more caring and supportive (Niculescu et al., 2013) and reduced frustration and stress (Hone, 2006). This consequently results in higher levels of engagement (Klein et al., 2002) and comfort (Bickmore and Schulman, 2007). Interestingly, in a high customer contact setting, service robots were found to have outperformed humans while performing standardised tasks. This was primarily due to the analytical and mechanical nature of social robots (Reis et al., 2020). However, when it comes to empathetic activities, social robots haven’t reached their full maturity (Reis et al., 2020). Thus, it is predicted that higher level of empathy will result in higher levels of emotional engagement (See below for H1d).

### Entertainment

This is the additional dimension introduced for the SERVBOT framework. Entertainment is defined as “activities that people enjoy and look forward to doing” (Harold Vogel in Entertainment Industry Economics, Cambridge University Press 1990). Entertainment engages users and is recognised as one of the strongest antecedents which lead to individuals’ satisfaction (Wakefield and Baker, 1998). If customers are entertained, they are more engaged and have longer interactions (Coulter et al., 2012; Liu et al., 2019). Since Sony’s entertainment robot “Aibo” was first launched in 1999, the world of robotics has seen the massive value in using robots as an “entertainment tool”. Businesses realise that when a customer is entertained, he or she spends greater time in that situation and is more likely to spend more on purchases (Christiansen et al., 1999). Additionally, consumer’s emotional engagement is the core strategy in the adoption of humanoid robots (Langen and Heinrich, 2019). In hospitality, service robots are employed to provide basic information to guests and entertain them (Mele et al., 2020). Thus, entertainment value is predicted as a driver of emotional engagement.

Previous research has demonstrated that in addition to the functional use of a technology, users also evaluate the entertainment value attached with the technology (Kim and Forsythe, 2008). Social robots possess permanent entertainment features (Anselmsson, 2016), such as their physical appearance, facial expressions, gaze and tone of voice (Aaltonen et al., 2017), and controlled, dynamic movements (Kuroki, 2001). Robots may also carry with them temporary entertainment features (Elmashhara and Soares, 2020) when they are customised to being part of a particular context. For instance, as a concierge for this study, Pepper (humanoid robot) was designed to narrate jokes such as, “The student asked if there was a shortcut to the train station as he/she was in rush. Pepper replied “Based on my calculations this is the fastest route to the train station. If you run really fast, you can get there in 1.5 min. I’ve done it myself and it’s a very good exercise.” Thus, higher level of entertainment value will result in higher levels of emotional engagement (See H1e below).

### Emotional Engagement

Marketers explain “engagement” as being related to an individual’s level of involvement and absorption in an activity (Seligman, 2012 cited in Di lascio et al., 2018), while the computing literature defines it as ‘the act of being occupied or involved with an external stimulus’ (Cohen-Mansfield et al., 2009). Across both disciplines, it is recognised that “engagement” is a psychological state (Patterson et al., 2006) and the study proposes that in the current context it represents a fundamental component of a person’s experience either with a social robot or a related activity (Monkaresi et al., 2017; Lascio et al., 2018).

There is a growing interest in the study of emotional engagement in the Human-Robot Interaction (HRI) field. Previously, the term “emotional engagement” has been interchangeable with emotional communication (e.g., Cohen-Mansfield et al., 2009) and emotional relationship (Ogawa and Ono, 2008). The study argues that emotional engagement is conceptually distinct from other concepts, such as communication and relationship. Emotional engagement is the amount of subconscious “feeling” experienced during an activity or an interaction (Heath 2009). Thus, the term “engagement” denotes an ongoing feeling over a longer timeframe. Researchers use a range of synonyms to describe the “emotional engagement”: involvement, passion, absorption, zeal, and dedication (Schaufeli, 2013). It is this internal state of an individual which provides the impetus to participate in certain behaviours (Finn and Zimmer, 2012). This study focuses on the emotional engagement dimension, which refers to the affective state (e.g., interest, happiness, pleasure) experienced by users while interacting with the technology (Schodde et al., 2017).

Previous studies have provided empirical evidence of a firm’s service quality influencing customers’ attitudes and behaviours (Zeithaml et al., 1996; Suh and Youjae, 2006; Al Azmi et al., 2012). A quality service enhances people’s experience with the organisation and leads to emotional engagement. Emotionally engaged customers spend more money (Sashi, 2012), are less price-sensitive, and are more likely to get through a problem than customers who are not so engaged. Such customers increasingly participate in co-creating value with the organization (Prahalad and Ramaswamy, 2004). When customers display high levels of emotional bonds with organisations, they develop affective commitment towards the company. Engaged customers are willing to go out of their way for a business and act as advocates. Such customers with high affective commitment are known to engage in word-of-mouth communication (Bowden, 2009; Harrison-Walker, 2001) and therefore help in building more business (Tripathi, 2014).

This type of engagement is more relevant for human-robot interaction (Hegel et al., 2008). Social robots are perceived by users as if they are real social actors. Social robots have anthropomorphic characteristics which helps elicit joy and sympathy (Hegel et al., 2008). Thus, robots bring with them a certain level of ‘social presence’ during human-robot interaction (Choi et al., 2014). This means that users may not notice the artificial nature of robots with whom they are interacting (Jung and Lee 2004). According to social impact theory (Latané, 1981), people are impacted by the real, implied, or imagined social presence of others. This psychological connection with another entity triggers a series of emotional responses such as a sense of personal, sociable, and warm human contact (Cyr et al., 2007). Thus, emotional engagement is important in how users experience their interaction with the robot. In fact, Huang and Alessi (1999) show people do not “think” about their experience with another social entity. In fact, they will *feel* it. The importance of “feelings” can be judged from the fact that “feelings” are unavoidable (Zajonc, 1980). Processing of emotions is fast and does not require conscious effort (Mast and Zalmter, 2006). Moreover, even if a person controls the expression of emotion, almost everyone will still experience the “feeling”.

**FIGURE 1:**
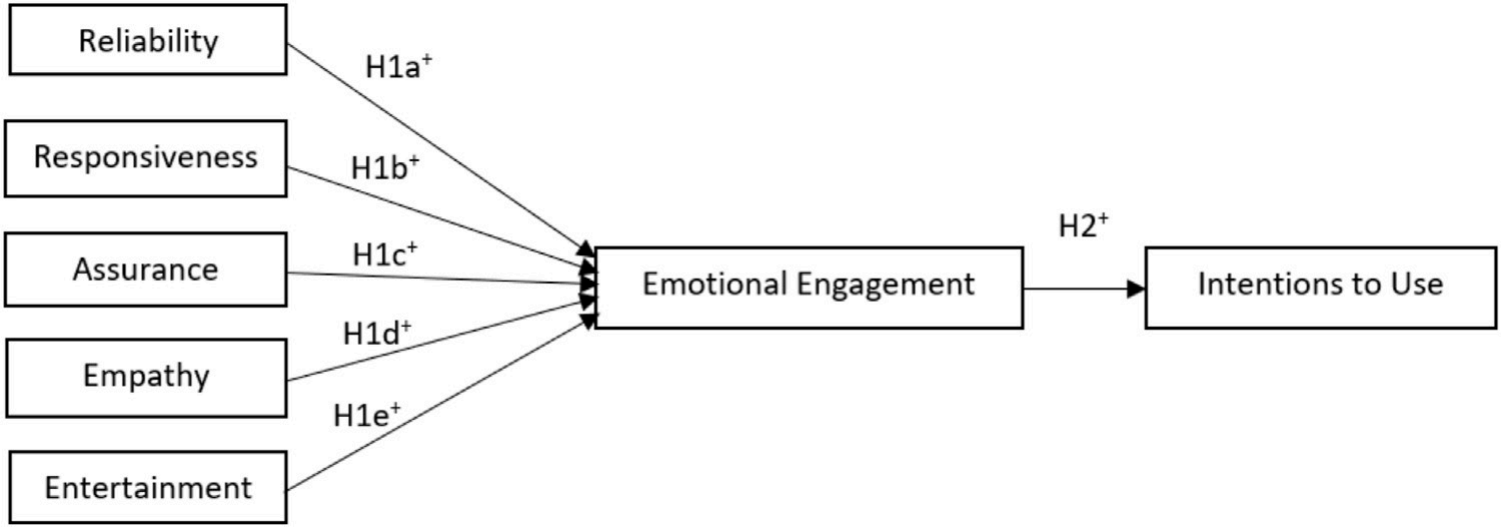
The Servbot model.

Based on these concepts the SERVBOT Model is theorised in Figure 1. This study explores the potential antecedents of engagement. The study hopes to identify the antecedents and other key variables in the Servbot model.

### Hypothesis 1a-e: SERVBOT Dimensions Will Be Positively Linked to Users’ Emotional Engagement With the Social Robot in a Service Setting

 H1a: Higher level of reliability will result in higher levels of emotional engagement.

H1b: Higher level of responsiveness will result in higher levels of emotional engagement.

H1c: Higher level of assurance will result in higher levels of emotional engagement.

H1d: Higher level of empathy will result in higher levels of emotional engagement.

H1e: Higher level of entertainment value will result in higher levels of emotional engagement.

### Emotional Engagement and Behavioural Intentions

When an individual is emotionally engaged in his/her interactions with a social robot, the person goes through a psychological process (Bowden, 2009). In this engaged state, the user is in “occupied, fully-absorbed or engrossed” (Higgins et al., 2009). Such levels of involvement promote a connection to the target object and others who may also be present during the interaction (Kahn, 1990). It is well-established in the literature that individuals who are emotionally engaged with an entity will not just be satisfied with their experience. In fact, more positive emotional experiences during a service interaction would result in delighting the user (Santos and Boote, 2003). Subsequently, this leads to more positive outcomes for the service provider. It has been empirically tested that once consumers are engaged with a brand, their emotive relationship has a direct impact on their intentions to undertake brand-related behaviours (e.g., Dwivedi, 2015). Based on these findings the study proposes:

H2: Higher level of emotional engagement with a social robot will result in greater behavioural intentions to use the robot.

## Research Methodology

The humanoid robot known as Pepper was used for this study. The robot is developed by Softbank Robotics and it is one of the most popular humanoid robots in the market (Softbank Robotics). The robot has 20 degrees of freedom for natural and expressive movements, and it supports speech and voice recognition. It also has a touch screen on its chest which is useful to display images and video clips.

### Pepper: A Social Humanoid Robot

The research study was conducted using Pepper robot, a social humanoid robot (Softbank Robotics). Pepper was used for two reasons. First, it’s the most widely used social robot for academic purposes (Pandey and Gelin, 2018). Secondly, Pepper is optimised for human interaction and can engage people through conversations and his touch screen. Additionally, Pepper does not fall into the Uncanny Valley Theory, and it is specifically designed to be a personal and service robot. It can exhibit body language, perceive and interact with its surroundings and move around (Pandey and Gelin, 2018). As mentioned earlier, gender and personality stereotypes impact how users perceive robots. Pepper is gender neutral and has androgynous and childlike voice, therefore the researcher is able to control for gender and personality variables. This eliminates stereotypes related to voice pitch, gender, culture and religious variables on service delivery. Further, Pepper can maintain eye gaze during face-to-face communication with the participants which enhances engagement. It can also hear sounds and turn its head to interact with the person speaking.

Additionally, Pepper can maintain eye gaze during face-to-face communication with the participants which also enhances engagement. When the participant’s start talking to Pepper, its eyes light up. It can also hear sounds and turn its head to interact with the person speaking. Pepper is equipped with facial recognition technology which helps it recognise faces and basic human emotions. It is a 1.2 m tall, wheeled humanoid robot. It’s 27 joints helps it move around smoothy and last for approximately 12 h at a stretch (Pandey and Gelin, 2018). It also has 20 degrees of freedom for natural and expressive movement along with speech recognition and perception modules helping it recognise and engage with the person. To enhance its functionality and usability, it comes equipped with a tablet attached to its chest that can help display and highlight important information. For example, when Pepper was deployed at the concierge desk, it was able to use its tactile head and hands along with eye gaze to engage with the user. It also has four microphones to help provide sound localisation. These natural multimodal interactions are integral to successful deployment of robots in human environments (Pandey and Gelin, 2018). Pepper is also known as an “emotionally intelligent” robot because of its ability to detect emotions and respond appropriately using its latest voice and emotional recognition algorithms (Engel, 2018; Pandey and Gelin, 2018). To make it safer for human use, there are no sharp edges, and its size and appearance makes it appropriate for a public space human-robot interaction (Pandey and Gelin, 2018).

### Scenario

This study employed a descriptive research design (“*social robot concierge condition*”). This study was approved by the Western Sydney University Ethics Committee (project code H13082). Qualtrics online survey was used to collect the data. A pool of undergraduate students was asked to complete the questionnaire after a casual interaction with the robot concierge. This group was selected as they are more likely to engage with social robots than other groups (De graaf and Allouch, 2013). By limiting respondents to the same “life stages” (in this case students) the researcher can control and reduce the external factors that may influence their decision (Silfver, 2003). The respondents were invited to interact with the robot during the in-class activity. Students did not receive any incentive to participate in the study and participating students had not interacted with a social robot earlier. Student participants were told to imagine that the service robot was at a concierge desk. The robot was placed at the front of the class and students volunteered to interact with the concierge robot. Participants were provided with possible questions to ask the robot. The procedure was as follows:

1) Pepper was brough into a room by a research assistant where the students were present. The robot was placed at the front of the class.
2) The students were asked to imagine that the robot is at the concierge desk after which the students were then given an opportunity to ask a series of questions to the concierge robot.
3) Participants were provided with the possible questions to ask the robot. For example, “Where is the train station?”, “Where is the closest bus stop?”, “How do I access the lifts?”, “Where is the event?”, etc. These questions are typically asked at the concierge desk and Pepper was pre-programmed to answer these questions.
4) Students volunteered to come up to the make-belief concierge robot and ask questions. They were encouraged to provide honest responses and were told that there were no right or wrong answers, to ensure the participants did not provide socially desirable responses.
5) For the entertainment dimension, Pepper was designed to narrate jokes such as,
  Student: I am in a rush. Is there a shortcut to the train station?
  Pepper: Based on my calculations this is the fastest route to the train station. If you run really fast, you can get there in 1.5 min. I’ve done it myself and it’s a very good exercise.
6) Immediately after the interaction, the students completed the SERVBOT questionnaire including demographic information (see Table 1 below). The online survey (see Table 2 below) consisted of five SERVBOT dimensions (reliability, responsiveness, assurance, empathy and entertainment value). The survey took about 10 min to complete.

**TABLE 1:** Respondent’s profile.

Age	Gender	Marital status	Occupation	Household income
18–24 (84%)	Male (46%)	Single (87%)	Student (88%)	A$0–A$7,999 (29%) A$7,800–A$15,599 (13%) A$15,6000–A$20,799 (13%)
	Female (54%)			

**TABLE 2:** Survey structure.

Survey structure
1. Scenario
2. Robot’s Service Quality (SERVBOT items)
3. Emotional Engagement and Behavioural Intentions
4. Demographics

## Results

A total of 94 respondents participated in the study. Prior research in HRI (Baxter et al., 2016) informs us that the typical sample size of studies in the HRI discipline are 30 subjects or less per condition. However, the researchers also undertook the test for the adequacy of sample size–KMO. The KMO measure of sampling adequacy indicates the proportion of variance in variables that might be caused by underlying factors (IBM, 1989). For example, a high value (close to 1.0) indicated that a factor analysis might be beneficial for the data whereas values than 0.50 indicate that factor analysis won’t be very useful (IBM, 1989). All resulting scores indicated that the sample size was sufficient for carrying out the required analysis. The demographic profile of the respondents is shown below.

All items were derived from the original SERVQUAL framework in the marketing literature (Parasuraman et al., 1988). Items for the “emotional engagement” were taken from a well-cited study (Fredricks et al., 2004). Four items for the “responsiveness” and “empathy” dimensions were reverse-coded and one for the “emotional engagement”.

Exploratory Factor Analysis (EFA) and Cronbach Alpha tests were conducted to ensure the measures were valid and reliable (see Table 3). Following the EFA results, one item for the ‘empathy’ dimension needed to be removed. The results for the remaining items in SERVBOT were found to be satisfactory. All Cronbach’s Alpha scores were above 0.7, the items measuring the dimensions are shown to be reliable.

**TABLE 3:** Factor analysis and reliability test.

Variable	Factor loading	KMO	Reliability (Cronbach’s alpha
Reliability		0.821	0.868
Pepper provides timely services	0.867		
Pepper appears to be smart and reassuring	0.823		
Pepper is capable of doing tasks in time	0.801		
Pepper is dependable	0.689		
Responsiveness		0.815	0.849
I do not think Pepper can perform well at the concierge (reverse-coded)	0.864		
Pepper does not provide good service (reverse-coded)	0.863		
I do not think Pepper can help customers (reverse-coded)	0.817		
Pepper is inarticulate when responding to people (reverse-coded)	0.783		
Assurance		0.674	0.772
I can trust Pepper	0.914		
I feel safe with Pepper	0.816		
Pepper can do a good job as the concierge	0.554		
I think Pepper is polite	0.420		
Empathy		0.757	0.761
Pepper does not have my best interests at heart (reverse-coded)	0.791		
Pepper is not available when customers need it (reverse-coded)	0.811		
Pepper does not know what my needs are (reverse-coded)	0.700		
Pepper does not give me personal attention (reverse-coded)	0.686		
Pepper provides caring and individualised attention to customers^a^	0.578		
Entertainment		0.853	0.965
Pepper is enjoyable	0.973		
Pepper is pleasing	0.942		
Pepper is entertaining	0.926		
Pepper is fun to use/watch	0.896		
Emotional Engagement		0.844	0.903
I felt happy watching Pepper the robot	0.897		
I felt excited by Pepper the robot	0.896		
I liked hanging out with Pepper the robot	0.803		
I am interested in the work being done by Pepper the robots	0.881		
I felt bored with Pepper the robot (reverse coded)	0.790		

a Item removed from the final analysis.

**FIGURE 2:**
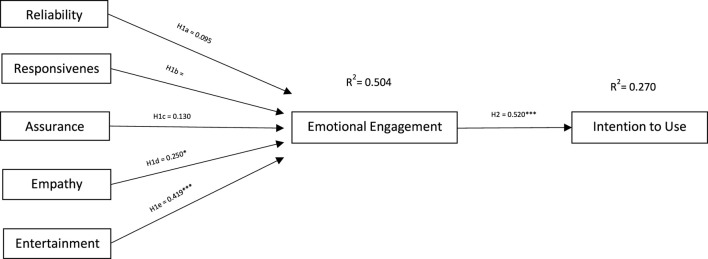
The Servbot model.

Multiple regression analysis was used to test the hypotheses for the SERVBOT model (Figure 2). Regression results show a positive and significant link between “*empathy”* (B = 0.226) and “*entertainment”* (B = 0.375) and *emotional engagement* (See Figure 2). Thus, H1d and H1e were accepted and other hypotheses were rejected for H1. Other SERVBOT dimensions did not demonstrate a significant link with users’ emotional engagement. As expected, results also indicate a strong, positive link (B = 0.520) between *emotional engagement* and *intention to use* the robot at the concierge desk (See Table 4). This is in line with the past research (Valentini et al., 2018; Alnsour and Al Faour, 2020; Kamboj et al., 2020) Therefore, H2 was also accepted. See Figure 2 and Table 4 for the summary of the results.

**TABLE 4:** Multiple regression.

Variables	Regression (beta)	*R* ^2^	t	Sig
		0.504		
Reliability → Emotional Engagement	0.095		1.014	0.313
Responsiveness → Emotional Engagement	0.047		0.485	0.629
Assurance → Emotional Engagement	0.130		1.333	0.186
Empathy → Emotional Engagement	0.250*		2.634	0.010
Entertainment value → Emotional Engagement	0.419		4.604	0.000
Emotional Engagement → Intention to use	0.520	0.270	5.833	0.000

Dependent variable: Emotional engagement *significant at 0.000.

Dependent variable: Intentions to use *significant at 0.000.

These results are partly in line with past research, the results indicate that robots’ service quality is predominantly driven by their ability to show empathy (Niculescu et al., 2013; Fung et al., 2016) and to be entertaining (Morita et al., 2020). On the other hand, three dimensions (reliability, responsiveness and assurance) of service quality were not linked to emotional engagement. This extends on the current literature and suggests that while these three SERVQUAL dimensions are important in influencing customer satisfaction, they have limited impact as SERVBOT dimensions in influencing emotional engagement.

The “reliability” of a service provider is viewed as an underlying factor leading to engagement. However, in the case of the social robot being reliable does not translate into a state of emotional engagement for the users. Interestingly, this phenomenon has also been highlighted in a previous study (McLachlin, 2000). Thus, users might find Pepper robot to “*be capable of doing tasks in time*”, but that may not be sufficient to get concierge-users “involved” or “fully absorbed” in the interaction and drive emotional engagement. For example, the findings suggest the robot’s ability to perform the task does not drive emotional engagement. That is, the robot is “expected” to complete the task efficiently.

Similarly, the responsiveness of the service provider (i.e., helping customers and responding to their needs or requests) is logically linked to customer engagement behaviours, such as positive word-of-mouth (Roy et al., 2020). However, in the study, the responsiveness of the robot is not linked to an emotional engagement with the respondents. Similar to the previous hypothesis, the robot is “expected” to be responsive. And the results indicate this does not drive emotional engagement as it is considered as completing a “task”.

“Assurance”, measured *courtesy* and *safety* related to the robot was also not found to have a significant impact on our participants’ engagement levels. This could be because the use of social robots is still new and customers focus on the empathy and engagement features more than just service quality. Additionally, customers could change their perceptions, views, and responses towards the robots after a prolonged interaction. For example, the customer’s first interaction with Alexa might find it as exciting but the same interaction over a long term would become common and thus, not receive much attention (Lu et al., 2020). However, the study anticipates when the novelty wears off, assurance could become the key dimension in service quality. To create a less alienating and more human-like experience, social robots will have to be reliable, human-like, responsive, assuring, empathetic, and entertaining. The extent to which social robots can excel at these will be the determining factor in their adoption and acceptance.

### Empathy and Emotional Engagement

Previous research shows emotional engagement is driven by being empathy and it is often treated as a dependent variable in a service interaction context (Leite et al., 2011). Context is important as empathy is “an ability to understand a person’s emotional reaction with the context” (Deutsch and Madle, 1975). If social robots are employed as concierge, then they will expected to interact with humans in empathetic way and possess the same capabilities as the human concierge (Niculescu et al., 2013). Empathy is integral to successful human-robot interactions as it facilitates the creation and maintenance of social relationships (Paiva et al., 2018). Moreover, it is important for social robots to understand the users emotions and also share their own, just like Pepper did in this study (Paiva et al., 2018). Literature has shown that social robots that have human-like features are perceived as more sociable and are easier to connect with emotionally (Kim et al., 2013).

For a social robot, being empathetic is essential to emotionally engage customers. Since social robots are treated as another social entity, it is easier for participants to emotionally connect with them during interactions. Empathy is a key component of engagement (Björling et al., 2020) and in this study, participants found Pepper to be empathetic. The reason for this could be that Pepper provided individualised attention to the participants and provided accurate responses to the participants questions. Further, after answering questions, Pepper asked the participants if there is anything else it could help them with, showcasing that it cared for the participant needs. Pepper has advanced voice recognition which meant it understood the questions asked by all the participants clearly and provided them correct answers. Additionally, it added “Hope that helps” after answering a question showcasing that it truly wanted to help the participant. Pepper made the participant feel at ease when the participant asked a simple question “I am really sorry but how do the elevator work?“. It did so by responding that it was not a problem and lots of people ask it this question followed by “Hehe” sound at the end of the sentence. For a customer this is important as the concierge should not only be able to provide a good service but also be polite, friendly and possess an appropriate sense of humour (Niculescu et al., 2013). The interaction finished with Pepper saying “I am glad that I can help. If you need anything else, I am always here from 6am to 10pm. You have a good day”. This information made the participant feel cared for and consequently, they “felt” happy and excited about interacting with Pepper. Front-line employees’ helpfulness or willingness to spend extra time and effort helping the customer is a cause of delight; customer delight further forms the basis on which front-line employees’ performance is assessed (Brady and Cronin, 2001). If a social robot is able to demonstrate its affective capability and reflect empathic behaviours such as listening or responding appropriately, it creates the scene for building a rapport with the user and this is a key antecedents of emotional engagement (Gaytan and McEwen, 2007).

Interestingly, a longitudinal study conducted by Gockley et al. (2005) indicated that even though many users interacted daily with the robot, after a certain time period, only a handful of them interacted with the robot for more than 30 s due to lack of engagement capabilities (Gockley et al., 2005). Post this study, the same robot was made more engaging by including proper greeting and farewell behaviours, more interactive dialogues, the ability to display emotions, and the ability to identify repeat visitors (Leite et al., 2013). In short, making the robot more engaging resulted in longer interactions by frequent visitors especially when the robot was in a negative mood. This is supported by the common ground theory (Leite et al., 2013). These interactions also depended on the user’s familiarity with the robot (Leite et al., 2013).

### Entertainment and Emotional Engagement

Entertainment had a significant influence on emotional engagement. This is expected as entertainment depends upon generating an emotional engagement with audiences, whether it be laughter, tear or thrills. Emotional engagement is also called involvement, and involved customers add meaning to entertainment products (Martin, 1998). This facilitates increased enjoyment (Neale, 2010). Essentially, the reason why customers consume entertainment is because of the pleasure they derive from doing so (McKee et al., 2014). Entertainment products are experience products that have symbolic value and customers engage with social robots for the experience (McKee et al., 2014).

Pepper has specifically been employed for entertainment purposes and as a concierge. Additionally, consumer’s emotional engagement is at the core of the strategy of using humanoid robots (Langen and Heinrich, 2019). Thus, entertainment is used to emotionally engage customers. Embodied or humanoid robots encourage customers to be more sociable and bond with them (De Gauquier et al., 2021).

In this study, Pepper entertained the participants by conversation and non-verbal cues. It used jokes along with gestures to keep the participants entertained and engaged during the interaction. This is in line with the previous research as customers expect social service robots to entertain them and retailers expect them to engage customers in social interactions (Niemelä et al., 2017). This study further adds to the literature as previous researchers have called for studies to examine entertainment and interactive scenarios between the robot and the customer (Aaltonen et al., 2017). The scenario in the study applied this method and it incorporated the use of jokes to keep the participants entertained. The original assessment tool–SERVQUAL–does not include entertainment as a dimension of service quality. However, being entertaining is a key characteristic for social robots, especially in retail settings (Aaltonen et al., 2017). In view of this observation, this study confirms the importance of ‘entertainment’ in the SERVBOT model.

### Emotional Engagement and Intentions to Use

As expected, emotional engagement is strongly linked with users’ intentions to use the robot for concierge services. Previous research in the use of technologies has demonstrated that when product usage engages participants, they view the technology as original and innovative, and it triggers intrinsic motivations (Shen and Eder, 2009). Intrinsic motivation has a deeper impact as it helps change the perceptions of users. It is also effective in bringing about a long-lasting behaviour change (Lee and Doh, 2012). Thus, emotionally engaged users are more likely to use technologically oriented products in the future as well. Previous studies have shown that emotional engagement is a predictor of behavioral intention, for example, emotionally engaged customers spend more money (Gallup Consulting 2009 cited in Sashi 2012).

It is worth noting that the concept of “engagement” has been defined in different ways in different contexts in the literature (Pansari and Kumar, 2017). The study’s conceptualisation of engagement is not in terms of positive actions of the users, but more in line with participants’ emotional connection with the robot. This offers a possible explanation of why some dimensions of service quality are not significant in driving emotional engagement. This provides a key contribution to the literature and suggests that there is a significant difference between SERVQUAL and SERVBOT. For the robots to be emotionally engaging the robot developers need to focus on empathy and entertainment.

## Discussion

The findings of this study have implications for service providers and designers who are looking at employing social robots to undertake frontline tasks. The research has highlighted and confirmed aspects of a robot-delivered service that generate emotional engagement. In the traditional service setting with a human-delivered service, five dimensions of service quality are assessed. However, in the case of social robots as a service provider, being empathic and entertaining is more important to emotionally engage with the customer.

Empathy is not a new topic in the human-robot interaction domain. Due to a social robot’s humanoid form, researchers have long been interested in measuring robots’ level of empathy, as perceived by users. Previously, there have been unrealistic expectations around the expectation of benefits from using social robots (Pino et al., 2015). The findings demonstrate that in the context of performing frontline tasks in a concierge setting, customers are emotionally engaged due to the robot being empathetic and entertaining (Čaić et al., 2019). If a social robot is able to demonstrate its affective capability and reflect empathic behaviours such as listening or responding appropriately, it creates the scene for building a rapport with the user (key antecedents of emotional engagement) (Gaytan and McEwen, 2007). Affective responses are related to the feeling of excitement which may mediate the connection between service quality and brand loyalty (Yüksel and Yüksel, 2007). It has been established that service quality is positively related to brand loyalty which is why service providers try to harness brand equity and loyalty by improving the service design and the customer experience (Prentice and Wong, 2016). When customers come in contact with favorable service experience, they react by evoking short term arousal and affective spirit. Service providers try to evoke customer senses and emotional valence through affective responses (Luo et al., 2019).

Literature has identified that interpersonal interactions with the frontline employees are critical to a customer’s evaluation of service quality (Babakus et al., 2009; Homburg et al., 2009). Since they are the first human contact, sometimes even last, their interaction with the customer creates a critical impression of how the service experience is going to be (Payne and Webber, 2006). Dagger et al. (2013) found that increasing the interpersonal skill levels of just frontline employees also increase customer perceptions of service quality (Dagger et al., 2013). Additionally, characteristics related to a person such as empathy, politeness and similarly are also important in building trust. The intangible nature of services such as politeness, friendliness, sensitivity and empathy along with the relational interaction between the customer and the front line employees are critical determinant of customer satisfaction (Dagger et al., 2013). One way of developing emotional bond and relational rapport with customers is by developing friendly relationships with them (Liu et al., 2016). This can help build long-term customer satisfaction. Since social robots are now being used in the frontline service setting, they will also need to possess high interpersonal skills. This is explained by the Uncanny Valley Theory, it suggests that humans treats robots as another social entity and apply same rules to their interactions as they would in human-human interactions. The results from this study supports this phenomenon and indicates that a positive relationship exists between empathy and emotional engagement.

Interestingly, the robot does not have to display its cognitive capabilities to emotionally engage users, this suggests that customers expect the robot to be efficient with completing the task. The study did not compare between high service quality vs a low service quality. However, the findings suggest that future studies should examine how social robots perform in high service quality vs a low service quality (e.g., 5-star hotel concierge vs. a 2-star hotel congeries). It is predicted that under these conditions, reliability, responsiveness and assurance may perform differently.

The relationship between entertainment and engagement has long been recognised as the key in the adoption of social robots (e.g., Karat et al., 2002; Coulter et al., 2012; Schodde et al., 2017; Liu et al., 2019). Further, studies in advertising have indicated that positive mood does not always generate positive evaluations (Yan et al., 2013). Therefore, it was interesting to see entertainment value was able to create positive mood and in turn generated positive evaluations. The original assessment tool–SERVQUAL–does not include entertainment as a dimension of service quality. However, being entertaining is a key characteristic that is used in robots, especially in retail settings (Aaltonen et al., 2017). In view of this observation, this study confirms the importance of “entertainment” in the SERVBOT model. Shopping malls and retail outlets compete on providing a range of entertainment activities (Lotz et al., 2010). Despite a comprehensive review of entertainment activities (Elmashhara and Soares, 2019) not many business researchers in retail marketing have included robots as potential entertainers. There is an inherent gap in the literature and currently, no study has conceptualised “entertainment” as a component of service quality. Entertainment is strongly linked to customers’ positive emotions, which play a critical role in the enactment of consumption-related behaviours, such as purchases (Kim and Ko, 2010; Jung et al., 2011). Thus, experiential services, which provide a hedonic experience, customers will not just make a cognitive evaluation of the service (e.g., reliability, responsiveness and assurance) but evaluate the entertainment value of the experience. Emotions have a critical role in forming an overall assessment of a service. It is worthwhile for marketers and social robot designers to use robot-enacted entertainment to trigger positive emotions. In addition, the introduction of the “entertainment” dimension to measure SERVBOT is another major contribution to the literature.

## Limitations and Future Research Directions

Like other studies in the field, there are methodological and implied limitations within the study. The study is limited to only one setting (university campus) with undergraduate students. Therefore, future studies should explore the use of social robots with other demographics. In addition, cultural aspects are worthy to be considered as some countries are less receptive to having robots in customer service roles as compared to others. For example, certain Eastern Asian countries have been known to be more accepting of social robots whereas European countries seem to be less receptive to robot-provided services (Lu et al., 2020).

This study focused on service quality provided by a specific type of social robot (e.g., Pepper). Future studies should compare the use of other types of social robots in other service industries (e.g., restaurants, hotels, airports, etc.). In addition, this study did not focus on the appearance of Pepper which might also affect the participant opinions.

Moreover, this study was based on the perception of a sample of students at a given point in time. The students have never seen or interacted with Pepper, and the novelty effect may have influenced the results. Thus, future studies should conduct a longitudinal study to control for the novelty effect and track consumer perceptions of SERVBOT over time. Young consumers are more accepting towards technology and they are more accepting towards robots. Thus, future studies should compare the perceptions of the social robot’s service quality between different age groups. It would also be interesting to see if the study can be generalised beyond large urban centres. The study also did not consider task complexity, future studies should compare SERVBOT in high complex tasks such as tertiary teaching vs low complex tasks such as information desk (or 5-star hotel concierge vs 2-star hotel concierge). Future studies should conduct experiments comparing Human-Robot Interaction (HRI), Human-Human-Interaction (HHI), and Human-Human and Robot-Interaction (HHRI) to validate the model. This will provide further validity to the SERVBOT model.

The study investigated service quality using SERVBOT dimensions and found empathy and entertainment value are the key to driving emotional engagement. Consequently, emotional engagement has a significant impact on future intention to use the social robot in a service setting. Therefore, the SERVBOT model proposes a theoretical model that could be used to measure social robot’s service quality. This provides businesses with opportunities to track the quality of the robot’s service delivery over time. Thus, the study suggests that low complex tasks such as providing information at the concierge desk can be completed by robots like Pepper (e.g., customers are likely to use the robot in the future at the concierge desk).

Future studies should also attempt to understand the perception of the more vulnerable population such as seniors or children in similar or different service settings. Service failure should also be investigated in a real-life scenario, whether customers will be more satisfied or less satisfied with the robots after they have encountered a service failure as compared to a frontline service employee.

This study is the first to propose a SERVBOT model for social robots and researchers should not overestimate the first insights into service robots. It is critical to use a widely accepted service model to measure service quality (e.g., SERVQUAL) so that researchers and business managers can track the performance of the service robots. A longitudinal study should be conducted to track changes in customer’s perception of robots’ service quality and thereby, affecting their intentions. Further testing of the SERVBOT is needed to ascertain the validity of the model. Nevertheless, this study has provided a strong theoretical foundation on how the social robot’s service quality could be measured.

## Data Availability

The raw data supporting the conclusions of this article will be made available by the authors, without undue reservation.
